# Preparation, Characterization, and Pharmacological Investigation of Withaferin-A Loaded Nanosponges for Cancer Therapy; In Vitro, In Vivo and Molecular Docking Studies

**DOI:** 10.3390/molecules26226990

**Published:** 2021-11-19

**Authors:** Hamid Saeed Shah, Usman Nasrullah, Sumera Zaib, Faisal Usman, Ajmal Khan, Umar Farooq Gohar, Jalal Uddin, Imtiaz Khan, Ahmed Al-Harrasi

**Affiliations:** 1 Institute of Pharmaceutical Sciences, University of Veterinary and Animal Sciences, Lahore 54000, Pakistan; hamid.saeed@uvas.edu.pk; 2 Institute of General Pharmacology and Toxicology, Goethe University Frankfurt am Main, 60596 Frankfurt am Main, Germany; nasrullah@em.uni-frankfurt.de; 3 Department of Biochemistry, Faculty of Life Sciences, University of Central Punjab, Lahore 54590, Pakistan; 4 Department of Pharmaceutics, Faculty of Pharmacy, Bahauddin Zakariya University, Multan 66000, Pakistan; faisal.usman@bzu.edu.pk; 5 Natural and Medical Sciences Research Center, University of Nizwa, Nizwa 616, Oman; ajmalkhan@unizwa.edu.om; 6 Institute of Industrial Biotechnology, Government College University, Lahore 54590, Pakistan; dr.mufgohor@gcu.edu.pk; 7 Department of Pharmaceutical Chemistry, College of Pharmacy, King Khalid University, Abha 62529, Saudi Arabia; jalaluddinamin@gmail.com; 8 Department of Chemistry and Manchester Institute of Biotechnology, The University of Manchester, 131 Princess Street, Manchester M1 7DN, UK

**Keywords:** withaferin-A, nanosponges, cancer therapeutics, flow cytometry, drug release, cell cycle, DNA fragmentation

## Abstract

The rapidly growing global burden of cancer poses a major challenge to public health and demands a robust approach to access promising anticancer therapeutics. In parallel, nanotechnology approaches with various pharmacological properties offer efficacious clinical outcomes. The use of new artificial variants of nanosponges (NS) as a transporter of chemotherapeutic drugs to target cells has emerged as a very promising tool. Therefore, in this research, ethylcellulose (EC) NS were prepared using the ultrasonication assisted-emulsion solvent evaporation technique. Withaferin-A (WFA), an active ingredient in *Withania somnifera*, has been implanted into the nanospongic framework with enhanced anticancer properties. Inside the polymeric structure, WFA was efficiently entrapped (85 ± 11%). The drug (WFA) was found to be stable within polymeric nanosponges, as demonstrated by Fourier transform infrared (FTIR) spectroscopy and differential scanning calorimetry (DSC) studies. The WFA-NS had a diameter of 117 ± 4 nm and zeta potential of −39.02 ± 5.71 mV with a polydispersity index (PDI) of 0.419 ± 0.073. In addition, scanning electron microscopy (SEM) revealed the porous surface texture of WFA-NS. In vitro anticancer activity (SRB assay) results showed that WFA–NS exhibited almost twice the anticancer efficacy against MCF-7 cells (IC_50_ = 1.57 ± 0.091 µM), as quantified by flow cytometry and comet tests. Moreover, fluorescence microscopy with DAPI staining and analysis of DNA fragmentation revealed apoptosis as a mechanism of cancer cell death. The anticancer activity of WFA-NS was further determined in vivo and results were compared to cisplatin. The anticancer activity of WFA-NS was further investigated in vivo, and the data were consistent to those obtained with cisplatin. At Day 10, WFA-NS (10 mg/kg) significantly reduced tumour volume to 72 ± 6%, which was comparable to cisplatin (10 mg/kg), which reduced tumour volume to 78 ± 8%. Finally, the outcomes of molecular modeling (in silico) also suggested that WFA established a stable connection with nanosponges, generating persistent hydrophobic contacts (polar and nonpolar) and helping with the attractive delayed-release features of the formulation. Collectively, all the findings support the use of WFA in nanosponges as a prototype for cancer treatment, and opened up new avenues for increasing the efficacy of natural product-derived medications.

## 1. Introduction

Natural chemicals produced from plants have historically been a significant source for medical drug discovery, and have produced numerous leads for the delivery of anticancer medicines [1]. Natural products or natural compounds obtained directly from plants account for about half of the chemotherapeutic biomolecules authorized by the Food and Drug Administration (FDA) [2]. The scientific and research community is increasingly focused on naturally produced compounds, which are believed to have less harmful side effects than contemporary therapeutic methods [3].

The plant genus *Withania*, which belongs to the Solanaceae (nightshade) family, has been utilized in traditional medicine in Southeast/Southwest Asia for centuries, including in the Unani and Ayurvedic systems, among others. Known by many other names, including Ashwagandha, Indian Winter cherry, and Indian Ginseng, *Withania somnifera* is one of the most prominent plants that is widely used to improve both physical and mental health [4,5]. Among various chemical constituents of the *Withania* genus, the withanolides are a group of naturally occurring C28-steroidal lactone triterpenoids, especially Withaferin A (WFA), the most potent withanolide found in Ashwagandha, and is responsible for a range of health-promoting actions on the body [6]. The anti-cancer effects of WFA have been shown in a wide range of cancer cells, including glioblastoma, neuroblastoma, multiple myeloma, leukemia, as well as breast, colon, ovarian, head and neck cancers [7,8,9,10]. The anti-tumor activity of WFA is not completely understood, but it appears to involve a variety of polypharmaceutical effects, including targeting cytoskeleton structure and the proteasome system, regulating the activity of heat shock proteins, reactive oxygen species (ROS)-mediated anticancer activity, inhibition of nuclear factor kappa B (NF-kB) and oncogenic pathways [11,12,13].

Cancer is a major public health problem worldwide, and is the second leading cause of death after ischemic heart diseases worldwide [14]. Due to ongoing demographic and epidemiological transitions, the global burden of cancer is rapidly increasing [15]. Asia constitutes roughly 60% of the world’s population (4.5 billion) and is responsible for nearly one half of new cancer cases and more than one half of cancer deaths worldwide [16]. The increase in the regional burden of cancer is largely a result of socioeconomic growth and the increasing size and aging of the population [17,18]. Among all cancer types, breast cancer is the fourth most common of all cancers, and occurs predominantly in women [19]. The incidence of breast cancer is increasing not only in developed countries, but this burden is substantially shifting to vulnerable populations in developing countries as well [20].

Therefore, researchers are confronted with a major challenge: how to deliver drugs to particular locations with pinpoint accuracy [21]. Recent advancements in nanotechnology have made it possible to synthesize and manipulate materials on the nanoscale [22]. One of these cutting-edge nanomaterials is the nanosponge (NS), which has holes of nanoscale dimensions [23]. The possible applications of nanosponges include medication delivery, the transport of biocatalysts and gases, the immobilization of enzymes, and the adsorption of harmful chemicals [24]. Nanosponges may be used to deliver both lipophilic and hydrophilic medicines [25]. Most drugs have porous exterior surfaces that allow for a regulated release of the medication [26]. The increased solubility and bioavailability of the medication lowers the adverse effects and allows for more precise control over drug distribution [27]. Currently, the utilization of nanosponge drug delivery systems in chemotherapy has emerged as one of the most promising areas of life science [28].

Therefore, in view of the aforementioned findings, we showcase the formation of WFA-encapsulated nanosponges exhibiting a strong effect on the suppression of breast cancer growth in comparison to cisplatin. The WFA nanosponges have been fully characterized using various techniques. In vivo and in silico methods have also reinforced the in vitro results. Collectively, the proposed approach will reduce the dosage requirement of WFA, resulting in the minimization/elimination of associated side effects.

## 2. Results and Discussion

### 2.1. Physical Characterization

#### 2.1.1. Differential Scanning Calorimetric (DSC) Analysis

**Figure 1:**
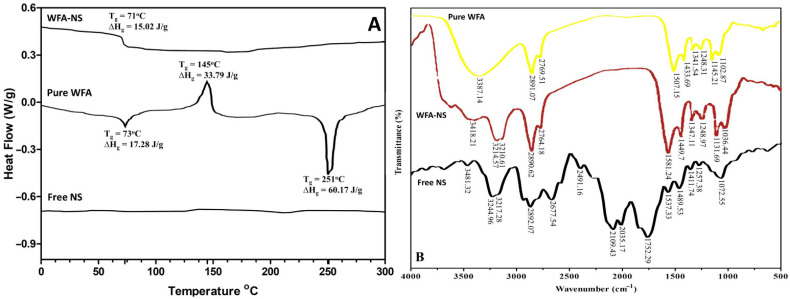
The representation of DSC thermogram of WFA-NS, pure WFA, and free NS (**A**), and FTIR spectra of pure WFA, WFA-NS and free NS (**B**).

The DSC thermogram offers useful details about the thermal properties, structural variability, and interactions (if any) between the therapeutically active agent and excipients [29]. To find compatibility among WFA and excipients (EC and PVA), the DSC thermogram was registered for pure WFA and WFA-NS. The DSC thermogram of pure WFA showed three peaks (Figure 1A). The first high corresponded to an endothermic peak (∆Hg = 17.28 J/g) with a 73 °C glass transition temperature (Tg). The second peak indicated an exothermic reaction (∆Hc = 17.28 J/g) with a crystallization temperature (Tc) of 148 °C. The third peak exhibited an endothermic reaction with a distinct melting point peak (Tm = 253 °C). Due to the absence of a melting point peak for WFA in WFA-NS, it was hypothesized that WFA will turn from crystalline to the amorphous or disordered crystalline phase within NS cavities. This circumstance verified the transformation of WFA from its crystalline to the amorphous state in NS [7]. Because of a rise in internal energy and a reduction in thermodynamic stability, this conversion may enhance the solubilization of the medication without affecting its physicochemical or pharmacological properties [30,31].

#### 2.1.2. Fourier Transform Infra-Red (FTIR) Spectroscopic Analysis

Figure 1B depicts the Fourier transform infrared spectra of WFA, Free NS and WFA-NS. It was discovered that the unique band produced by O-H stretching in the WFA spectrum was located at 3387.14 cm^−1^, and O-H stretching caused a significant peak (3418.21 cm^−1^) in the WFA and NS spectra as well. A strong peak was detected in the WFA at 2891.07 cm^−1^ as a result of C–H alkane stretching vibrations, and a similar peak was also seen in the WFA-NS at 2890.62 cm^−1^. Further within the functional group region, the WFA spectrum revealed a peak at 2769.51 cm^−1^, which was also seen in the WFA-NS spectrum, caused by C=O stretching (2764.18 cm^−1^). When the WFA peak (1507.15 cm^−1^) of C–H alkane bending vibrations was moved to a higher wavenumber (1581.24 cm^−1^) in WFA-NS, it was discovered that a redshift had occurred. In addition to the bending vibration of the C–H alkane group at 1433.69 cm^−1^, a bathochromic change was observed in the spectrum of WFA–NS (1449.7 cm^−1^). Having an alkoxy group (C–O) in the fingerprint region produced a peak at 1145.21 cm^−1^ in the spectrum of WFA. This peak was also present with a minor change in the spectrum of WFA-NS at 1131.69 cm^−1^, indicating that the alkoxy group was present in the fingerprint area. As a result, the functional groups of WFA remained unchanged, implying full connection between pure WFA and its NS counterpart Furthermore, the Free NS (ECNS) spectrum revealed characteristic peaks (3481.32, 3244.96, and 3217.28 cm^−1^) that were also observed in the WFA-NS spectrum with a minor shift, indicating that there was no chemical interaction between the polymer (EC) and the drug (WFA).

#### 2.1.3. Scanning Electron Microscopic (SEM) Analysis

**Figure 2:**
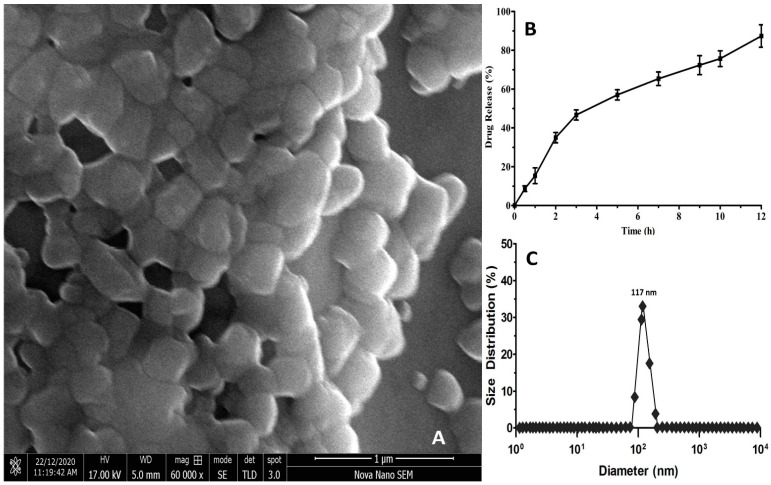
Scanning electron microscopic analysis of WFA-NS (**A**), WFA release from NS (**B**), and hydrodynamic diameter of WFA-NS (**C**).

Other than surfactants, the additives in NS may be critical in enhancing the physical characteristics of the substance. PVA has been the most extensively studied additive that is used to enhance the NS porous structure [32]. The porous nanostructure of NS (Figure 2A) produced through the ultrasonication assisted-emulsion solvent evaporation technique reinforced the earlier findings [33,34,35].

#### 2.1.4. Estimation of Nanosponges Hydrodynamic Diameter

In the prepared WFA-NS, a reasonable hydrodynamic diameter of 117 ± 4 nm (Figure 2C) and a high zeta potential value were found, as well as a credible estimate of the polydispersibility index (0.419 ± 0.073), as reported in Table 1. The zeta potential of dispersed particles is controlled by their Brownian motion, and larger zeta potential is linked with their higher dispersion stability [36]. The electrostatic stabilization on the NS surface was confirmed by the zeta potential measurements, which revealed a considerably negative value of −39.02 ± 5.71 mV [37]. A PDI represents particle size distribution within a sample that may be used to evaluate whether the dispersion is homogenous (≤0.3) or heterogeneous (>0.3) [38,39]. The produced WFA-NS had a PDI value within an acceptable range (0.389 ± 0.091), and if it surpassed 0.7, the DLS research could not be conducted owing to the high degree of variability in the size distribution [40].

#### 2.1.5. Drug Release Kinetics Studies

Figure 2B illustrates the findings of WFA release from NS in a regulated manner. To further understand the process of WFA release from NS, the collected data were correlated into kinetic models using the DDsolver program, which was then evaluated. Table 1 displays the values of the regression coefficients for each model. WFA was shown to be released over a long period in an in vitro release trial (12 h). Moreover, the evidence of in vitro release was examined utilizing pharmacokinetic designs [41]. A regression model (R^2^) containing 0.9806 indicated that drug particles were equally distributed in the NS matrix, which was more consistent with the Higuchi model than the previous findings [42,43]. It was discovered that the regression coefficient (R^2^) values derived from first-order (0.9867) and Korsmeyer-Peppas (0.9713, *n* = 0.324) models demonstrated dose-dependent release behavior, which was substantiated by the Fickian type of diffusion [44].

#### 2.1.6. Entrapment Efficiency (EE)

According to the findings described in Table 1, WFA demonstrated a good entrapment efficiency (85 ± 11%), suggesting that the drug was adequately encased in the NS. Typically, EE of a drug needs adjustment of several formulation parameters to keep the medication in a sponge-like structure.

Since WFA is a water-insoluble drug, it exhibits increased drug-polymer interaction and miscibility in organic solvent (DCM), ensuring maximal entrapment in nanocarriers. This finding was consistent with the previous report, indicating that EC nanosponges may be suitable for encapsulating the hydrophobic drug WFA [45]. On the other hand, boosting the entrapment effectiveness of nanosponges by increasing EC content enhanced the solution’s viscosity. Increased viscosity led to the creation of a thick polymer network, which prevented the medication from escaping the matrix [35,46].

### 2.2. Pharmacological Characterization

#### 2.2.1. Anticancer Activity (SRB Dye Assay)

The total protein generated when cells were treated with putative anticancer drugs was assessed using a very precise calorimetric technique. A negative charge fluorescent dye, sulforhodamine B (SRB), is a water-soluble luminous dye that electrostatically binds to proteins when the pH of the medium is significantly lowered. It can readily connect with cells that have been fixed with trichloroacetic acid (TCA) [47]. A bright color is produced by the SRB dye when it binds solely to intracellular proteins, and the color is proportional to the quantity of protein present in the cell. SRB performance was shown to be dependent on the concentration, with the weakest activity (less color) observed at higher concentration levels and the greatest activity (more color) detected at lower inhibitor concentrations [48].

**Figure 3:**
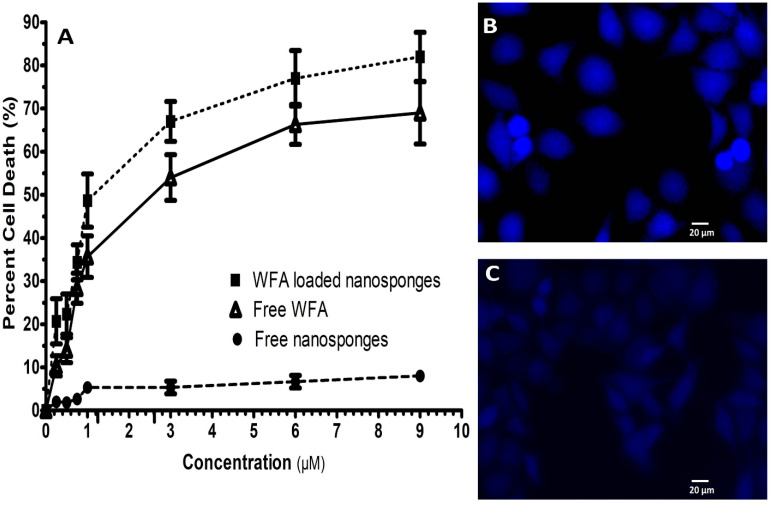
The pharmacological characterization of WFA-NS, pure WFA, and free NS (IC_50_ data) (**A**), DAPI staining of untreated and treated MCF-7 cells (**B**,**C**).

The initial screening revealed that WFA and WFA-NS had an IC_50_ of 3.41 ± 0.134 µM and 1.57 ± 0.091 µM, respectively, whereas empty NS was inert (Figure 3A) [49]. Our results were consistent with earlier research, emphasizing the importance of medication delivery through NS in contrast to its pure form [50,51].

#### 2.2.2. DAPI Staining

In DAPI staining of WFA-NS treated MCF-7 cells, apoptotic bodies with denatured cell membranes were seen, whereas untreated MCF-7 cells revealed no aberrant signals (Figure 3B,C respectively). DAPI staining is a qualitative study that identifies morphological alterations in the cell nucleus, which can help in the detection of apoptosis [36]. The nuclei of the untreated MCF-7 cells were consistent in size and shape with smooth edges. However, DAPI staining revealed fragmented and contracted nuclei in the treated cells. Our findings were consistent with previous studies [37].

#### 2.2.3. Genotoxicity Assessment

**Figure 4:**
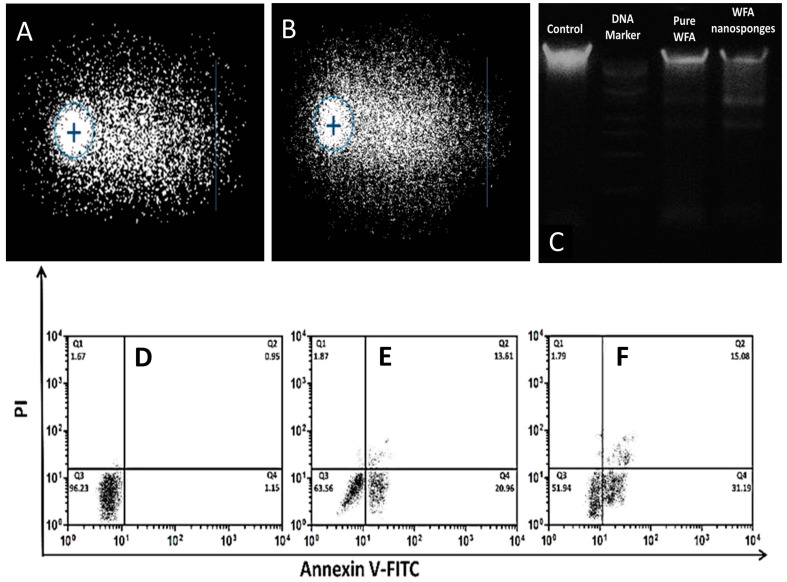
A representation of alkaline comet assay H_2_O_2_ (**A**), WFA-NS (**B**). DNA fragmentation analysis utilizing 1 Kb DNA ladder, pure WFA and WFA-NS (**C**). The flow cytometry analysis of untreated (**D**) pure WFA (**E**) and WFA-NS (**F**) against MCF-7 cells.

To validate any symptoms of DNA damage in a quantitative manner, single-cell gel electrophoresis (SCGE) or the comet assay were utilized [29]. A total of 100 cells per slide were counted and analyzed on comet tail length. The amount of DNA damage was determined by measuring the difference in genetic material between the nucleus (comet head) and the tail [30,31]. In the alkaline comet experiment, the percentage of DNA in the tail of WFA-NS was 56.70%, which was comparable to 61.38% in the tail of the comet treated with standard H_2_O_2_ (Figure 4A,B) [52].

#### 2.2.4. DNA Fragmentation

The results of DNA fragmentation analysis were used to back up the findings of the staining procedures. DNA from treated MCF-7 cells was run on a 1% agarose gel and evaluated using a gel documentation system. The findings revealed that the chemicals tested produced DNA fragmentation in MCF-7 cells. To assess the degree of fragmentation with test chemicals, a 1 kb DNA ladder was utilized as a marker, and cisplatin was used as a benchmark drug.

The pure WFA and WFA-NS revealed a fragmented DNA pattern. WFA-NS induced DNA fragments that were cleanly separated and showed no evidence of necrosis while pure WFA did not initially yield obvious fragments, but later on a clear fragment was seen. The presence of DNA fragments in sample lanes proved that cancer cells died as a result of WFA-induced apoptosis (Figure 4C).

#### 2.2.5. Flow Cytometry Analysis

Apoptosis is a biological suicide operation that clears the body of unwanted cells. Membrane modification, chromatin material shortening, and the formation of apoptotic bodies are all morphological changes seen in apoptotic cells [35].

Following prior qualitative studies that confirmed cancer cell death because of apoptosis, a flow cytometry study was performed to quantitatively validate the earlier results. Pure WFA and WFA-NS produced a significant amount of apoptotic cell death in treated MCF-7 cells (31.19%), while pure WFA caused only 20.96% apoptosis (Figure 4E,F) (*p* < 0.05). In MCF-7 cells treated with WFA-NS, the higher proportion of necrotic cells may be ascribed to better drug penetration or a longer duration of action induced by the controlled-release of NS formulation [53,54].

### 2.3. Animal Studies

#### In Vivo Studies

**Figure 5:**
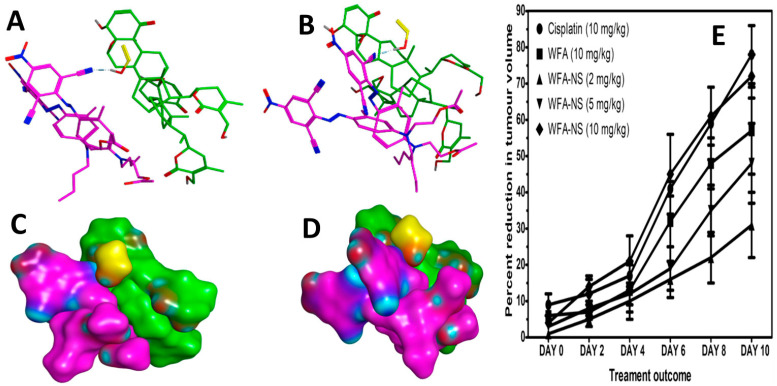
Molecular details of nanosponges assembly (**A**,**B**). The single polyvinyl alcohol (yellow stick) molecule mediates hydrogen bonding with the ethylcellulose (magenta sticks) (**A**). The results of molecular modeling studies, the bottom panels showed the MS–MS surface view of nanosponges (**C**,**D**). In vivo anticancer studies conducted on mice (**E**) with 10 mg/kg of Cisplatin (●) 10 mg/kg of pure WFA (■), 2 mg/kg of WFA-NS (▲), 5 mg/kg of WFA-NS (▼), and 10 mg/kg of WFA-NS (♦). The WFA-NS formulation exhibited a substantially lower relative tumor volume than free WFA (*p* < 0.05).

Thus far, we have shown that WFA-NS may substantially enhance the cytotoxicity of WFA against tumour cells in vitro. To support our findings, an in vivo experimental methodology was developed using Swiss Webster female albino mice, and the results were compared to cisplatin, a standard anticancer drug (Figure 5E). Table 2 reports the data related to the growth of tumor mass in experimental and comparison groups, which includes pure WFA, WFA-NS, and cisplatin (positive control) [55].

As anticipated before, groups A and B of the tumor-bearing mice administered WFA and free NS failed to inhibit tumor development. Treatment groups C and D, which contained cisplatin and pure WFA, respectively, showed a 78 ± 8 and 57 ± 12% reduction in tumor volume. These did not contradict the previously published data comparing the anticancer potential of WFA to that of cisplatin [56,57,58]. Several dilutions of WFA-NS were prepared with WFA concentrations that corresponded to 2, 5, and 10 (mg/kg) of mice in each of the E, F, and G treatment groups in order to assess the efficacy of a delivery mechanism in comparison with pure medicine (WFA). At Day 10, the intervention group G had a decrease in tumor volume to 72 ± 6%, which was 15% greater than the pure WFA therapy group (57.12%) at the same time. Our findings support prior research efforts that explain the importance of using a nano-drug delivery method to provide a rapid treatment response in a variety of disorders [59,60,61].

### 2.4. In Silico Studies

#### Molecular Docking Studies

Molecular docking studies were carried out to illustrate the structure of the WFA-NS by utilizing the default docking protocol in MOE [62]. Initially, the structure of WFA was developed. The lowest energy models were analyzed visually to comprehend the molecular basis of interaction between constituents of nanosponge assembly. Figure 5C,D demonstrated an MS–MS surface diagram of the WFA-NS complex. The hydrophilic contribution by the hydroxyl group of WFA and the polar head of ethylcellulose was laid at the poles of the nanosponges. Figure 5A,B exhibited the details of molecular interactions stabilizing the complex. A hydrogen bond was seen between polyvinyl alcohol and ethylcellulose. The hydroxyl groups of the polyvinyl alcohol molecule depicted hydrogen bonds with ethylcellulose that had a long aliphatic chain draped against the steroid ring system. This observation was in line with the previous report [63]. WFA embedded in the nanosponges has disclosed anticancer and cytotoxic activity for an extended period in comparison to pure WFA. Molecular modeling revealed that the WFA created a stable assembly with the nanosponges. It is speculated that in an aqueous environment, these interactions between drugs and polymeric surfactants lead to sustained hydrophobic contacts and delayed the release of the drug.

## 3. Materials and Methods

### 3.1. Preparation of Withaferin-A Loaded Nanosponges

WFA-loaded nanosponges were prepared by ultrasonication assisted-emulsion solvent evaporation technique (ESE-Tech) [64,65]. Briefly, 200 mM ethyl cellulose (EC) and 100 mM WFA were dissolved in dichloromethane (20 ML) to make the organic phase. An aqueous phase containing 2 mM PVA was prepared separately in 50 mL of deionized water. Subsequently, the organic phase was emulsified in a dropwise manner into the aqueous phase over 15 min (50 s on-off cycles) using sonication at an elevated frequency (>2.5 kHz). PVA was employed to prevent the NS from clumping. Furthermore, the dispersion was stirred at 1000 rpm for 24 h using a thermostatically controlled magnetic stirrer. The WFA-NS were then rinsed three times with ultra-pure water to eliminate any adsorbed PVA. Finally, the WFA-loaded NS were extracted by centrifugation (40,000× *g*, 20 min) and stored at 4 °C until further use.

### 3.2. Characterization of Withaferin-A Loaded Nanosponges

#### 3.2.1. Differential Scanning Calorimetric (DSC) Analysis

Thermal testing of the pure WFA, free NS and WFA-NS was performed to show the physical stability of the WFA in NS. The sample was heated at a rate of 10 °C/min with a constant nitrogen supply (2 mL/min) to avoid oxidation. After heating each sample to 300 °C, the DSC scan was performed on each sample and tested against an empty aluminum pan on DSC 214 Polyma (NETZSCH Instruments, Burlington, VT, USA).

#### 3.2.2. Fourier Transform Infra-Red (FTIR) Spectroscopic Analysis

FTIR spectra of WFA, free NS and WFA-NS were obtained using KBr disk technique. The test sample was mixed with KBr powder to generate the KBr disc. The IRTracer-100 FTIR spectrophotometer ((Shimadzu IRPrestige-21, Tokyo, Japan) was used to measure the FTIR spectra (4000 to 400 cm^–1^).

#### 3.2.3. Scanning Electron Microscopic (SEM) Analysis

The SEM analysis was performed on a Hitachi S-4700 (Houghton, MI, USA) with 10–20 kV acceleration voltage. Using ethanol, the sample was rapidly disseminated and applied on freshly cleaned silicon wafers to dry. Likewise, the specimens were gold-sputter-coated in order to improve the conductivity of the material.

#### 3.2.4. Estimation of Nanosponges Hydrodynamic Diameter

The size distribution of NS was determined by using dynamic light scattering. For DLA investigation, 5 mg of every lyophilized formula was suspended in ultra-pure water. The zeta potential was measured using the Malvern Zetasizer Nano ZS90 (Cambridge, UK).

#### 3.2.5. Study of Drug Release Kinetics

A diluted WFA-NS (100 mM WFA) in PBS (10 mL, pH 7.4) was transferred to a dialysis bag, which was submerged in PBS (500 mL) with lysozyme (1.2 µg/mL) to emulate the in vivo environment. The trial included a magnetic stirrer (75 rpm, 37 °C). The spectrophotometer detected the release of WFA at a predetermined time and the collected data were analyzed using release kinetic models to ascertain the inside mechanics of WFA release from NS with the help of the DDsolver tool.

#### 3.2.6. Entrapment Efficiency

For each formulation, the entrapment efficiency (EE) was determined by using the previously reported technique, with minor modifications [66]. WFA-NS formulation (2 mL) was infused onto a dialysis membrane and spun for 1 h at room temperature on a magnetic stirrer (100 rpm). A UV-Visible spectrophotometer was used to measure the absorbance of the sample, which was determined to be 218 nm. To compute the EE, the following equation was used:

Entrapment Efficiency (% EE) = (WFA entrapped in NS)/(WFA added in NS) × 100(1)

#### 3.2.7. Anticancer Activity (SRB Dye Assay)

The anticancer activity of pure WFA and WFA-NS was tested on human breast cancer cells (MCF-7 cells) using the sulforhodamine B (SRB) assay [67]. The cells were seeded in a 96-well plate (1 × 10^4^ cells per well) and allowed to develop for 24 h. To test the anti-cancer effects, different quantities of WFA and WFA-NS were tested. Free NS and cisplatin were used as negative control and standard anticancer agent, respectively. Following a 24 h incubation with the materials, the cells were fixed for 1 h using 40% ice-cold trichloroacetic acid (TCA). Subsequently, the cells were rinsed with PBS and allowed to dry in the open air. Fixed cells were stained with SRB dye at 0.4% (*w*/*v*) for 30 min at room temperature. Finally, each 96-well was added 100 µL of 10 mM Tris Base (pH 10.5), which was gently swirled for 5 min. The microplate absorbance reader was used to measure the absorbance at 565 nm using ELISA microplate reader ELx808™ (BioTek instruments, Winooski, VT, USA). Prism 5.0 was used to visualize the IC_50_ values (µM).

#### 3.2.8. DAPI Staining

The cells were grown in a two-well sterile chamber slide with 2 × 10^4^ cells per well. The growing cells were cultured with pure WFA and WFA-NS (dosage ≥ IC_50_) for two treatments. Triton X-100 solution was used to repair the cells (0.1%). After staining the cells with DAPI (10 µg/mL), they were allowed to rest in the dark for 10 min before repeating the process. The cells were then incubated in DAPI (10 µg/mL) for 10 min. The unabsorbed DAPI has washed away with PBS during multiple rinses. The cells were observed at certain wavelengths using a fluorescent microscope (Nikon Eclipse-Ni, Minato, Tokyo, Japan). The wavelengths for emission and excitation were 461 and 358 nm, respectively [68].

#### 3.2.9. Genotoxicity Assessment

A comet test was conducted for detecting double-strand DNA (dsDNA) breaking using a previously established protocol [69,70]. When MCF-7 cells were treated with WFA-NS, a suspension of 2 × 10^4^ cells/well was prepared. Comet slides were coated with a cell suspension that had been diluted with 1% low-melting-point agarose (LMPA). The slides were put in a lysis solution (10 mM Trizma-X, 10% DMSO, 2.5 M NaCl, 1% Triton-X, 100 mM EDTA) at pH 10. The samples were charged in horizontal electrophoresis tanks holding a buffer solution with pH 13 for the time-course experiment (300 mM NaOH and 1 mM EDTA). The DNA was untwisted inside an alkaline buffer. Thereafter, the slides were cleaned thoroughly with methanol and air-dried. CaspLab 1.2.3b2 tools were used to assess the comet’s DNA damage.

#### 3.2.10. DNA Fragmentation

The inhibitors were added on separate wells for 48 h according to their IC_50_ values. After trypsinization, the cells pellet was then re-dissolved in a DNA extraction buffer (10 mM Tris (pH 7.4), 10 mM EDTA, 0.5% Triton X-100) and incubated at 4 °C for 10 min. The mixture was spun in a centrifuge for an additional 30 min to facilitate the breakdown of the RNA molecule. When the lysate was treated with proteinase K solution at 50 °C, the sample degraded rapidly. The solution was treated with 0.5 M NaCl and 50% IPA and kept refrigerated overnight. DNA was solubilized using the Tris-EDTA buffer. One well had a 1 kb DNA ladder, whereas cisplatin was used as the reference drug. The control and test wells were kept equally on the gel. All DNA samples were electrophoretically examined and stained with ethidium bromide to detect fragmented DNA under UV irradiation [71].

#### 3.2.11. Flow Cytometry Analysis

WFA, WFA-NS, and cisplatin were incubated separately with MCF-7 cells (1 × 10^6^) for 24 h. Cells were trypsinized using the trypsin-EDTA solution at 37 °C for 5 min. The cell culture medium was gently introduced to avoid cell clumping. After being treated with 500 µM H_2_O_2_, the cells were reconstituted in 100 µL of binding buffer for 15 min. Subsequently, the cells were treated with annexin-V-FITC dye for 15 min before being placed in a dark environment. The pellet was further resuspended in 200 mL annexin-V binding buffer and stained with 5 μL of propidium iodide. The cells were tested using FACS with a 600 nm emission filter for PI and a 545 nm filter for annexin-V-FITC. Next, 10,000 cells were collected and then examined using the CytoFLEX Flow Cytometer (Beckman Coulter Life Sciences, Brea, CA, USA) [72].

#### 3.2.12. In Vivo Studies

Adult female albino BALB/c mice (25–30 g) were housed at the Faculty of Pharmacy, Bahauddin Zakariya University, Multan, Pakistan. The mice were housed at 24 °C with a 12 h light and dark cycle and were given normal rat chow with unrestricted water supply. Five animals were housed in a steel mesh cage to minimize the discomfort caused by overpopulation. Animals were treated following the rules established by the Faculty of Pharmacy, Bahauddin Zakariya University, Multan’s Ethics Committee on the Care and Use of Animals in Scientific Research (50/PHP/20). The mice were split into seven groups of five mice each at random. A total of 4 × 10^6^ MCF-7 cells were injected into the flanks of female mice from Groups B-G using an i.p injection technique. The tumor was left to develop to approximately 50 mm^3^ [55]. The treatment and control groups are summarized in Table 3.

From the second day following MCF-7 cell administration, the tumor volume was measured daily. The following equation was used to quantify the tumor volume of the mice:

Tumor volume = (Length × Width^2^)/2 (2)

While length is the longest dimension and width is the dimension that is perpendicular to the length. To evaluate the anticancer efficacy of each formulation, the tumor inhibition rate (TIR) was computed.

TIR (%) = [(Tumor weight of sample group)/(Tumor weight of the control group)] × 100(3)

#### 3.2.13. Molecular Docking Studies

The structures of PVA, ethycellulose, and WFA were obtained from PubChem. Briefly, the canonical SMILES were obtained and were converted to a three-dimensional structure using the Builder module in MOE [62]. The partial atomic charges were calculated, followed by energy minimization according to a steepest–descent protocol using the Merck Molecular Force Field (MMFF94X) in MOE with a Root Mean Square gradient of 0.01 Å. By the stoichiometric calculation of nanosponges, a single molecule of polyvinyl alcohol and two molecules of ethylcellulose were used for calculation. Furthermore, two molecules of WFA was docked against a single nanosponge assembly. The resulting poses were analyzed visually. All graphics were extracted using MOE and Discovery Studio software using previously reported protocols [73].

#### 3.2.14. Statistical Analysis

A paired *t*-test was used to analyze flow cytometry and genotoxicity studies. The level of significance was set at 5% (*p* < 0.05). Other research findings are provided as mean standard deviation (SD). Microsoft Excel (2010), SPSS (9.0), and Prism (5.0) Software was used to conduct the statistical analysis.

## 4. Conclusions

In summary, the distribution of natural anticancer compounds like WFA was successfully achieved inside the nanosponge structure. The WFA-NS were less than 120 nm in size, and WFA was embedded as an amorphous form, as revealed by DSC, with no change in the drug molecule’s chemistry. The FTIR and molecular docking studies had confirmed a stable complexation between WFA and EC inside the nanosponges. The medication was efficiently entrapped and released continuously from the NS for 12 h. The anticancer studies (SRB assay) revealed that the WFA-NS was more effective as compared to pure WFA with an IC_50_ value of 1.57 ± 0.091 µM and 3.41 ± 0.134 µM, respectively. In the comet assay, the WFA-NS cellular contact demonstrated significant DNA damage (56.70%), while standard H_2_O_2_ showed a slightly higher value (61.38%). Similarly, the WFA-NS showed more potential in killing cancer cells via apoptosis (31.19%) as compared to pure WFA (20.96%). A reduction in tumor volume was seen when tumor bearing mice were treated with WFA-NS (72 ± 6%), and the results were in good agreement to cisplatin activity (78 ± 8%). Collectively, the WFA-NS described here could serve as a prototype platform for natural materials such as cancer therapeutics, thereby expanding nutraceutical potential in chronic metabolic disorders.

## Figures and Tables

**Table 1:** WFA-NS physical and kinetic characteristics (Mean ± S.D, *n* = 3).

Analysis	Results
Diameter hydrodynamic	117 ± 4 nm
Entrapment Efficiency (%)	85 ± 11
Zeta Potential (mV)	−39.02 ± 5.71
Poly dispersity Index(PDI)	0.389 ± 0.091
Zero-order	0.8734
First-order	0.9867
Higuchi Model	0.9806
Korsemeyer Peppas, *n* value	0.9713, 0.324

**Table 2:** The numerical data provides percent reduction in tumor volume after giving doses (mg/kg of a mouse).

	Percent Reduction in Tumor Volume				
	Cisplatin (C) 10 mg/kg	WFA (D) 10 mg/kg	WFA-NS (E) 2 mg/kg	WFA-NS (F) 5 mg/kg	WFA-NS (G) 10 mg/kg
DAY0	9 ± 3	6 ± 1	1 ± 1	3 ± 1	4 ± 1
DAY2	12 ± 5	7 ± 2	5 ± 3	8 ± 2	14 ± 2
DAY4	17 ± 3	13 ± 8	10 ± 6	12 ± 3	21 ± 7
DAY6	41 ± 2	32 ± 11	16 ± 7	19 ± 6	45 ± 11
DAY8	59 ± 10	48 ± 7	22 ± 11	35 ± 7	61 ± 8
DAY10	78 ± 8	57 ± 12	31 ± 9	48 ± 11	72 ± 6

**Table 3:** The anticancer effects of WFA, WFA-NS, and cisplatin were shown in both experimental and comparison groups of mice.

Group	Type of Treatment
A	Water for injection in cancerous mice (WFI)
B	Free nanosponges (NS) for cancerous mice
C	Cancerous mice were given 10 mg/kg cisplatin
D	WFA (10 mg/kg) treated cancerous mice
E	WFA-NS (2 mg/kg) treated cancerous mice
F	WFA-NS (5 mg/kg) treated cancerous mice
G	WFA-NS (10 mg/kg) treated cancerous mice

## Data Availability

The data presented in this study are available from the authors on reasonable request.

## Sample Availability

Samples of the compounds (withaferin-A loaded nanosponges) are available from the authors.
